# High frequency of HPV genotypes 59, 66, 52, 51, 39 and 56 in women from Western Mexico

**DOI:** 10.1186/s12879-020-05627-x

**Published:** 2020-11-25

**Authors:** Andrea Molina-Pineda, María Guadalupe López-Cardona, Laura Patricia Limón-Toledo, Juan Carlos Cantón-Romero, María Guadalupe Martínez-Silva, Holanda Vanesa Ramos-Sánchez, María Guadalupe Flores-Miramontes, Pedro de la Mata-González, Luis F. Jave-Suárez, Adriana Aguilar-Lemarroy

**Affiliations:** 1 División de Inmunología, Centro de Investigación Biomédica de Occidente (CIBO)-Instituto Mexicano del Seguro Social (IMSS), Sierra Mojada No. 800, Col. Independencia, 44340 Guadalajara, Jalisco Mexico; 2 Programa de Doctorado en Ciencias Biomédicas, Centro Universitario de Ciencias de la Salud (CUCS), Universidad de Guadalajara, Guadalajara, Jalisco Mexico; 3 Unidad de Medicina Genómica y Genética, Hospital Regional Dr. Valentín Gómez Farías, ISSSTE, Zapopan, Jalisco Mexico; 4 Departamento de Fisiología, CUCS, Universidad de Guadalajara, Guadalajara, Jalisco Mexico; 5 Clínica de Displasias, Unidad Médica de Alta Especialidad (UMAE), Hospital de Ginecología y Obstetricia, Centro Médico Nacional de Occidente-IMSS, Guadalajara, Jalisco Mexico; 6 Servicio de Ginecología Oncológica, UMAE Hospital de Ginecología y Obstetricia, Centro Médico Nacional de Occidente, IMSS, Guadalajara, Jalisco Mexico; 7 Departamento de Anatomía Patológica, Centro Médico Nacional de Occidente-IMSS, Guadalajara, Jalisco Mexico

**Keywords:** HPV, Cervical cancer, CIN 1, Linear Array, Mexico

## Abstract

**Background:**

Human papillomavirus infection is an important factor associated with cervical cancer (CC) development. The prevalence and genotype distribution vary greatly worldwide. Examining local epidemiological data constitutes an important step towards the development of vaccines to prevent CC. In this work, we studied the prevalence of HPV genotypes in women from Western Mexico with the COBAS 4800 and/or Linear Array Genotyping Test (LA).

**Methods:**

The samples analysed in this study represent a population from Western Mexico, which includes six different states. Our approach was first to test for HPV in cervical samples from women who attended their health clinic for routine gynaecological studies (open-population, *n* = 3000) by utilizing COBAS 4800. Afterwards, 300 of the HPV-positive samples were randomly selected to be genotyped with LA; finally, we genotyped samples from women with cervical intraepithelial neoplasia grade 1 (CIN 1, *n* = 71) and CC (*n* = 96) with LA. Sociodemographic data of the diverse groups were also compared.

**Results:**

The overall HPV prevalence among the open-population of women as determined by COBAS 4800 was 12.1% (*n* = 364/3000). Among the HPV-positive samples, single infections (SI) with HPV16 were detected in 12.4% (*n* = 45/364), SI with HPV18 were detected in 1.4%, and infection with at least one of the genotypes included in the high-risk HPV pool was detected in 74.5% of the cases. LA analysis of the samples showed that in addition to HPV genotypes 16 and 18, there was a high prevalence of HPV genotypes 59, 66, 52, 51, 39 and 56 in women from Western Mexico. With respect to the sociodemographic data, we found statistically significant differences in the number of pregnancies, the use of hormonal contraceptives and tobacco intake.

**Conclusions:**

Our data indicate that there is a high prevalence of HPV genotypes which are not covered by the vaccines currently available in Mexico; therefore, it is necessary to include HPVs 59, 66, 51, 39 and 56 in the design of future vaccines to reduce the risk of CC development. It is also essential to emphasize that the use of hormonal contraceptives and tobacco smoking are risk factors for CC development in addition to the presence of HPV.

## Background

Cervical cancer (CC) is the fourth most frequently diagnosed cancer and the fourth leading cause of cancer death in women worldwide, resulting in an estimated 570,000 new cases and 311,000 deaths in 2018 [1]. In Mexico, CC is the third most common cancer in women; it has a high incidence (7869 new cases each year) and causes 4121 deaths each year according to Globocan 2018 [1]. It has been demonstrated that persistent infection with various human papillomavirus (HPV) genotypes plays a major role in the development of high- and low-grade cervical intraepithelial neoplasia (CIN) and CC [2–4]. To date, more than 220 HPV genotypes have been described [5], of which at least 40 genotypes infect the female genital tract [6, 7]. Based on the evidence observed in women with normal cytology, precursor lesions and CC, the International Agency for Research in Cancer (IARC) has classified genotypes 16, 18, 31, 33, 35, 39, 45, 51, 52, 56, 58 and 59 as carcinogenic to humans (Group 1). HPV68, on the other hand, has been classified as probably carcinogenic to humans (Group 2A), and genotypes 26, 53, 66, 67, 70, 73, 82, 30, 34, 69, 85 and 97 are classified as possibly carcinogenic to humans (Group 2B) [8, 9]. HPV infection is one of the most common sexually transmitted diseases in Mexico. Although this virus is present in more than 90% of CC cases, only a small proportion (≈ 1%) of infected women develop cancer [10].

Worldwide, HPV16 and 18 constitute approximately 75% of all cervical cancer cases. After HPV 16 and 18, HPV genotypes 31, 33, 35, 45, 52 and 58 are the most frequently found. Although their contribution varies by country and region, these HPV genotypes cause approximately 20% of cervical cancer cases [11, 12]. However, the prevalence and genotypic distribution of HPV infections varies greatly between populations; for example, HPV 31 and 33 are more prevalent in Europe and the United States, while genotypes 35 and 45 are more frequent in Africa and 52 and 58 in Asia [11]. Recent findings of our research group indicated that the most prevalent HPV genotype found in all diagnostic groups in different regions of Mexico (Monterrey, Guadalajara, Tepic, Mexico City, Metepec and Tlaxcala) is HPV16, followed by HPVs 62, 51/84, 18 and 53/Cp6108 in women without cervical lesions; HPVs 84, 58, 59 and 62 in low-grade CIN; HPVs 31, 18/70 and 6/51/59/66/Cp6108 in high-grade CIN; and HPVs 18, 45, 52/58 and 39 in CC samples [13]. Regional data on the prevalence and genotypic distribution of HPVs are essential for estimating the impact of vaccines on CC and screening programmes. The aim of this study was to estimate the prevalence of HPV genotypes among women undergoing routine gynaecological care in different regions of Western Mexico (Aguascalientes, Colima, Guanajuato, Jalisco, Michoacan and Nayarit). The results of the current study might help to estimate the effectiveness of currently available HPV vaccines and the development of screening programmes to prevent and decrease the incidence of CC in Mexico.

## Methods

### Cervical sample collection

Our study consisted of 3 groups of patients: 1) the open-population, 2) women with CIN 1 and 3) women with CC. The collection of the samples was conducted from 2015 to 2019. Open-population samples (*n* = 3000) were obtained from women seeking routine gynaecologic care at the Hospital Regional Valentín Gómez Farias (ISSSTE, Mexico), which receives samples from diverse states of Western Mexico, including Aguascalientes, Colima, Guanajuato, Jalisco, Michoacan and Nayarit. The precancerous lesion samples (*n* = 77) and cervical cancer samples (*n* = 96) were collected from women attending the Dysplasia Clinic and Oncology Service, respectively, at UMAE Hospital de Gineco-Obstetricia Centro Médico Nacional de Occidente (CMNO, IMSS). We exclusively included women who attended gynaecological services for the first time who did not have any prior treatment and whose diagnosis was confirmed by histopathology. Cases with insufficient data (without age or diagnosis confirmation) or poor DNA quality or quantity were excluded. Cervical samples were collected during gynaecological examination by inserting a cytobrush into the endocervical canal and rotating it for 3–5 full turns. Samples were placed in transport medium solution (Thin Prep PreservCyt® solution, Cat. No. 70097–002; Hologic, Bedford, MA, USA). All participants provided written informed consent prior to sample collection. All participant data were managed confidentially. This study was approved by the Ethics and Research Committees of the Instituto Mexicano del Seguro Social (IMSS), CONBIOETICA-09-CEI-009-20,160,601 with the registration numbers R-2014-785-036 and R-2018-785-147 and from the ISSSTE Research Committee (registration number ISSSTE/CEI/2013/080).

### HPV detection by COBAS 4800 test

Open-population samples (*n* = 3000) were analysed by the COBAS 4800 test (Roche Molecular Diagnostics, Pleasanton, CA, USA). The COBAS 4800 HPV test is an automated qualitative in vitro test for the detection of HPV DNA in patient specimens. The test utilizes amplification of target DNA by polymerase chain reaction (PCR) and nucleic acid hybridization for the detection of 14 high-risk HPV (HR-HPV) genotypes in a single analysis. The probe specifically identifies HPV16, HPV18 and a pool of 12 other HR-HPV genotypes (31, 33, 35, 39, 45, 51, 52, 56, 58, 59, 66 and 68) at clinically relevant infection levels. The COBAS 4800 HPV test was performed according to the recommendations of the manufacturer [14].

### HPV detection by linear Array (LA) genotyping test

HPV genotyping was conducted in 300 HPV-positive samples that were selected randomly and in CIN 1 and CC samples. Total DNA was isolated and purified from each cervical sample using the AmpliLute Liquid Media Extraction Amplicor Kit (Cat. no. 03750540 190; Roche Molecular Systems, Inc., Branchburg, NJ, USA). HPV-positive samples were genotyped by the Linear Array® HPV Genotyping test (Cat. no. 04391853 190; Roche Molecular Diagnostics, Pleasanton, CA, USA), which can simultaneously detect up to 37 HPV genotypes in a sample (6, 11, 16, 18, 26, 31, 33, 35, 39, 40, 42, 45, 51, 52, 53, 54, 55, 56, 58, 59, 61, 62, 64, 66, 67, 68, 69, 70, 71, 72, 73 (MM9), 81, 82 (MM4), 83 (MM7), 84 (MM8), IS39 and CP6108). This assay is based on PCR amplification of target DNA using PGMY-HPV universal primers, hybridization of the amplified product to oligonucleotide probes and their detection by colorimetric reaction. The probe includes β-globin amplification in the same PCR as an internal control. Detection and genotype determination were performed using the denatured amplified DNA and an array of oligonucleotide probes located in the L1 region that permitted identification of individual HPV genotypes. All procedures were carried out following the manufacturer’s instructions [15]. According to the results obtained by LA, HPV frequencies in each study group and the percentage of single- and/or multiple infections (SI or MI, respectively) of each HPV genotype were determined.

### Statistical analysis

A database was constructed for the results of each sample; it contained the genotypes detected in the samples, reported as the presence (positive) or absence (negative) of each studied HPV genotype detected by COBAS 4800 or Linear Array, the age of the analysed patient, age at menarche, age at first intercourse, pregnancies and use of hormonal contraceptives and tobacco. Data were analysed using IBM SPSS Statistics version 25.0 software (IBM Corp., Armonk, NY), and *p*-values< 0.05 were considered significant.

## Results

### Characteristics of the study patients

Sociodemographic characteristics such as age, age at menarche, age at first intercourse, pregnancies, use of hormonal contraceptives and tobacco consumption of the participants from the three different study groups were analysed (as shown in Table 1).

**Table 1:** Sociodemographic characteristics of the study groups participants

	Open-population (***N*** = 300) % (N)	CIN 1 (N = 71) % (N)	CC (N = 96) % (N)	***p***-value
***Age (years)***				
**16–25**	3.3 (10)	11.3 (8)	2.1 (2)	< 0.001
**26–35**	26.3 (79)	46.5 (33)	13.5 (13)	
**36–45**	28.3 (85)	21.1 (15)	31.3 (30)	
**46–55**	28.3 (85)	14.1 (10)	20.8 (20)	
**56–65**	10.7 (32)	7.0 (5)	17.7 (17)	
**66–75**	2.7 (8)	0 (0)	10.4 (10)	
**76–85**	0.3 (1)	0 (0)	4.2 (4)	
**Median**	43.0	33.0	47.0	
***Age at menarche***	12.7 ± 1.6	12.4 ± 1.8	12.7 ± 1.8	0.263
***Age at 1st intercourse***	20.4 ± 4.0	19.8 ± 4.9	18.6 ± 3.5	0.069
***Pregnancies***	2.5 ± 1.9	2.1 ± 2.0	4.4 ± 3.3	< 0.001
***Hormonal contraceptives***				
**Yes**	13.7 (41)	14.1 (10)	28.1 (27)	0.038
**No**	86.3 (259)	85.9 (61)	71.9 (69)	
***Smoker***				
**Yes**	12.3 (37)	8.5 (6)	34.4 (33)	< 0.001
**No**	87.7 (263)	91.5 (65)	65.6 (63)	

Data are presented as the mean ± SD. T-Student/Chi square tests were used to determine statistical significance between the means of CIN 1 and CC groups. *p*-values< 0.05 were considered statistically significant.

The participants ranged from 18 to 82 years old, and participants were analysed in 7 different groups according to their ages. Interestingly, in the open-population group, women aged 36–55 years showed the highest overall HPV positivity rate; in the CIN 1 and CC groups, the highest rates of HPV infection were observed in the 26–35 years and 36–45 years groups, respectively. We found a statistically significant difference in the average number of pregnancies, the use of hormonal contraceptives tobacco consumption between the CIN 1 and CC groups. The average number of pregnancies in the CC group was twice that observed in the CIN 1 group. Only 14.1% of the CIN 1 patients reported using hormonal contraceptives, whereas the use increased to 28.1%. in the CC group. On the other hand, only 8.5% of CIN 1 women were described as active smokers, while the percentage in the CC group presented a very noticeable increase to 34.4%.

### HPV detection by COBAS 4800 test

A total of 3000 female subjects seeking routine gynaecologic care at the family planning and gynaecological clinic, Hospital Regional Valentín Gómez Farías, ISSSTE, Mexico, were recruited. As shown in Tables 2, 2636 women (87.9%) were HPV negative, and 364 women (12.1%) tested positive for at least one HR-HPV detected by the COBAS 4800 test. For the 364 HPV-positive women, the positivity rates for single infection (SI) with HPV16 or HPV18 were 12.4 and 1.4%, respectively, whereas 74.5% of the samples were positive for at least one of the other HR-HPVs included in the pool. The prevalence of HPV16 or HPV18 together with any other HR-HPV of the pool was 8.8% or 3.0%, respectively.

**Table 2:** HPV prevalence in the cervical screening population in Western México

COBAS 4800 HPV Test	N	%
Negative	2636	87.9
Positive	364	12.1
Total	3000	100.0
COBAS 4800 Positive Samples	N	%
HPV16 (SI)	45	12.4
HPV16 + Other genotypes^a^	32	8.8
HPV18 (SI)	5	1.4
HPV18 + Other genotypes^a^	11	3.0
Other HPV genotypes^a^	271	74.5
Total	364	100

^a^ Other HPV genotypes include 12 HPV genotypes: 31, 33, 35, 39, 45, 51, 52, 56, 58, 59, 66 and 68. SI: single infection

### HPV genotyping by LA test

**Fig. 1:**
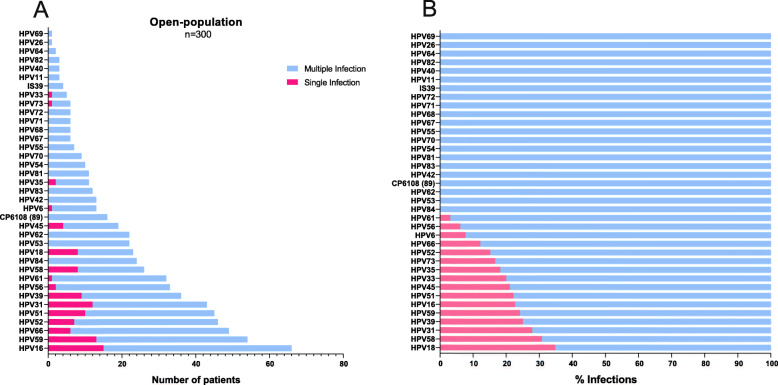
HPV genotypes detected by linear array (LA) in the open-population group. **a** The graph shows the frequency of HPV genotypes found by LA as single (pink bars) or multiple infections (blue bars). **b** The graph shows the percentages of single (pink bars) or multiple infections (blue bars) calculated for each individual HPV genotype, considering the number of samples in which a particular HPV was detected as 100%

From the 3000 patients included in the COBAS 4800 analysis, 300 HPV-positive samples were randomly selected to be genotyped by LA. As shown in Fig. 1a, all HPV genotypes included in this test were present in the open-population group. The most frequent HPV genotypes found were HPV16 (22.0%, *n =* 66), HPV59 (18.0%, *n =* 54), HPV66 (16.3%, *n =* 49), HPV52 (15.3%, *n =* 46), HPV51 (15.0%, *n =* 45), HPV31 (14.3%, *n =* 43), HPV39 (12.0%, *n = 36*) and HPV56 (11.0%, *n =* 33). On the other hand, HPV genotypes varied across the six different geographical regions, and the most common HPV genotypes by state are shown in Fig. S1 (Additional file 1). Regarding the HPV-positive samples of the open-population, we found that 32.7% (*n* = 98) had a single infection, and 67.3% (*n* = 202) had multiple infections. To better understand the percentage of cases with SI or MI of each HPV, an analysis considering 100% of the total number of positive samples for each genotype was performed. Thus, as shown in Fig. 1b, HPV genotypes such as 84, 53, 62, 89 (CP6108) and 42 (among others) were only found as coinfections; some HPV genotypes were also found as SI, but in a very low proportion, as shown for HPV 18, 58, 31, 39, 59, 16 and 51.

**Fig. 2:**
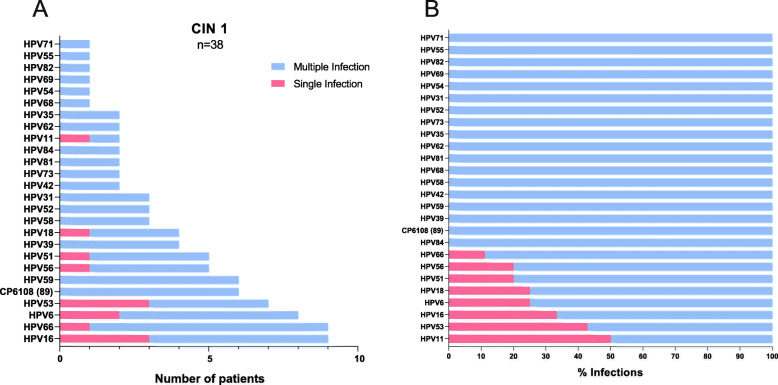
HPV genotypes detected by linear array (LA) in the CIN 1 group. **a** The graph shows the frequency of HPV genotypes found by LA as single (pink bars) or multiple infections (blue bars). **b** The graph shows the percentages of single (pink bars) or multiple infections (blue bars) calculated for each individual HPV genotype, considering the number of samples in which a particular HPV was detected as 100%

According to these results, it was of interest to determine whether the HPV genotypes detected in the open-population group and not included in the currently available vaccines were present in samples of women diagnosed with precancerous lesions or invasive CC. For this purpose, patient samples of both diagnosis groups were also collected and analysed by LA. As shown in Fig. 2a, 26 HPV genotypes were identified in the patients diagnosed with CIN 1, where HPV positivity was detected in 53.5% (*n* = 38/71) of the samples. In the positive samples, the HPV genotypes most frequently found in the CIN 1 group were HPV16 and 66 (23.7%, *n* = 9), HPV6 (21.1%, *n* = 8), HPV53 (18.4%, *n* = 7), HPV59/89 (CP6108) (15.8%, *n* = 6), HPV51/56 (13.2%, *n* = 5), and HPV18/39 (10.5%, *n* = 4). Of the 38 HPV-positive samples, 22.5% (*n* = 16) had SI, and 77.5% (*n* = 55) had MI. HPV genotypes such as 84, 89 (CP6108), 39, 59, 42 and 58 (among others) were only found in coinfections (as shown in Fig. 2b); in contrast, some genotypes were also detected as single infections, such as 11, 53, 16, 6 and 18.

**Fig. 3:**
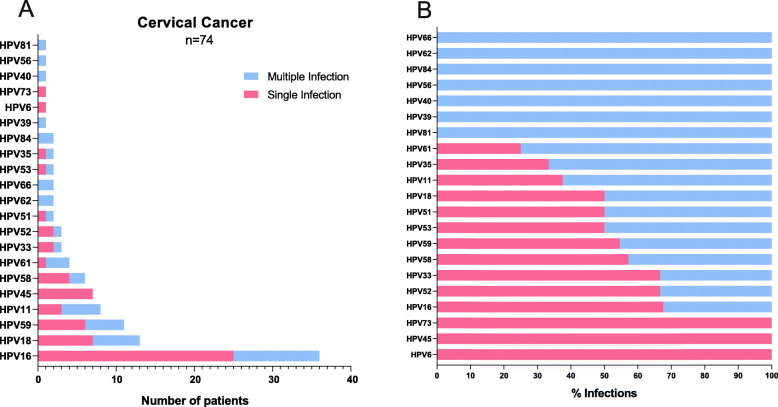
HPV genotypes detected by linear array (LA) in patients with cervical cancer (CC). **a** The graph shows the frequency of HPV genotypes found by LA as single (pink bars) or multiple infections (blue bars). **b** The graph shows the percentages of single (pink bars) or multiple infections (blue bars) calculated for each individual HPV genotype, considering the number of samples in which a particular HPV was detected as 100%

Finally, concerning the CC group, only 21 HPV genotypes were found (as shown in Fig. 3a). HPV positivity was detected in 77.1% (*n* = 74/96) of the samples; considering only the HPV-positive samples, the most commonly found HPV genotypes were HPV16 (50%, *n* = 37/74), HPV18 (18.9%, *n* = 14/74), HPV59 (14.9%, *n* = 11/74), HPV11 (10.8%, *n* = 8/74), and HPV45/58 (9.5%, *n* = 7/74). Concerning HPV coinfections, it was determined that 75.7% (*n* = 56) were present as SI and 24.3% (*n* = 18) as MI. In this group, the majority of the HPVs were found in a large percentage as SI (as depicted in Fig. 3b), and only HPVs 81, 39, 40, 56, 84, 62 and 66 were found as MI.

## Discussion

The role of HPV infections in the development of cervical cancer has acquired fundamental importance, and in recent years, HPV has become a relevant diagnostic and prognostic tool. Therefore, knowing the HPV genotypes that predominate in each country region is crucial.

In addition to HPV infection, different risk factors have been associated with the development of cervical cancer, such as early age at first sexual intercourse, number of sexual partners, high parity, education level, smoking habit, use of hormonal contraceptives and certain dietary deficiencies [16–21]. In our study, as depicted in Table 1, the number of pregnancies in women with CC was significantly higher than that in patients with CIN 1; different reports have shown that parity ≥3 is correlated with the risk of CC development. This result agrees with results from some [19, 22] but not all previous studies [23–25]. Consistent with results from previously published reports [26, 27], our study highlighted the use of hormonal contraceptives in a high percentage of women with CC (28.1%), while their use was lower in women with CIN 1 (14.1%). However, other studies did not find an association between the use of hormonal contraceptives and the risk of CC development [19, 24, 25]. In relation to tobacco consumption, smoking is one of the most studied cofactors for cancer development [28]. In our analysis, 34.4% of women diagnosed with CC claimed to be smokers compared to 8.1% of women with CIN 1. Although this cofactor has been associated with a two-fold increase in the risk of CC development [22], some authors suggest that tobacco intake is only associated with the risk of developing squamous cell carcinoma and not adenocarcinoma [29].

Focusing on HPV infections in the open-population among different regions of Mexico, 12.1% (364/3000) of women attending routine gynaecological examinations were found to be HPV-positive, with an overall prevalence of 2.6% for HPV16 (*n* = 77/3000), 0.5% for HPV18 (*n* = 16/3000) and 9.0% for other HPVs excluding 16 and 18 (*n* = 271/3000) (Table 2). Similar results, like those of the FRIDA study, have been previously reported in Mexico; this study found global HPV positivity of 11.0% in an open-population as determined by COBAS 4800, of which 8.8% corresponds to high-risk HPVs (excluding HPV16 and HPV18) [30, 31]. Different rates of infection have been recorded in diverse parts of the world. Studies in other regions have reported an overall HPV prevalence of 12.2% in Canada [32], 34.5% in Peru, 10.3% in Iran, and 9.3% in Australia [14, 33, 34]. On the other hand, Aoyama-Kikawa et al. reported the presence of HPV infection in only 4.6% of Japanese women [35]. These results support the findings of Becker et al., who showed regional and ethnic differences in HPV prevalence [36].

On the other hand, the most commonly detected HPV genotypes that we found by linear array in the open-population group were 16, 59, 66, 52, 51, 31, 39 and 56 (Fig. 1a). Although these genotypes have been detected in the open-population, we observed that some of them are also frequently present in the CC group, such as HPV 16, 59, 52, 51 and 66 (Fig. 3a). These genotypes have been classified by the IARC as carcinogenic to humans (except for HPV66, which is classified as “possibly” carcinogenic) [9], and there is sufficient evidence of their presence in different types of cancer, such as anus, vulva, vagina, penis and some head and neck cancers [37]. Nevertheless, the heterogeneity of the HPV genotypic distribution among states in Mexico is evident in this work (Fig. S1-Additional file 1). Expanding on the findings of each common genotype identified in this study, HPV16 was the most prevalent genotype found in all diagnostic groups, as previously reported in Mexico [13, 38–40] and worldwide [41, 42] by different authors. Interestingly, HPV59 ranks second in the open-population group, fourth in CIN 1 and third in CC, and it has been identified in previous studies among different populations of Mexico including Cozumel (an island in eastern Mexico), Monterrey (northern Mexico) and Michoacan (western Mexico) [43–45]. It is important to highlight that Salcedo et al. detected HPV59 in only 1% of CC samples from Mexican women [46]. Moreover, in a previous study by our research group using only the linear array HPV genotyping test, we also found a very low rate of HPV59 in cervical cancer (0.8%) [13]. In both previously mentioned studies, samples were obtained from Mexican women before 2014. In the present study, in which the samples were collected from 2015 to 2019, a dramatic increase in the prevalence of HPV59 was observed, reaching 11.5% when all CC samples were analysed (*n* = 11/96) (Fig. 3a). This HPV genotype has also been observed in the top five HPV genotypes detected in different regions of the world, as reported, for example, in Ghana [47], China [48–50], and Switzerland [51].

HPV66 is the third most common genotype that we detected in the open-population (16.3%); it was the second most common in CIN 1 (23.7%) and the ninth in CC (2.7%). HPV66 was recently reported to also have a high prevalence in low-grade cervical lesions of women from Mexico City (45.7%); the prevalence was lower in CC samples (3.6%), although they used a different detection method (INNO-LiPA) [52]. HPV66 has also been frequently found in Chile [53], Africa [54, 55] and China [56], among others.

HPV52 and 51 were also frequently found in this study; however, they were mostly detected as coinfections. Both genotypes have been commonly reported as single infections and also as coinfections; the presence of HPV51–52 and coinfections of three genotypes, such as HPV16–51-52, are frequently detected in coinfections [57, 58].

Otherwise, even though a positivity rate of HPV above 99% has been reported in cervical cancer [6, 59], we found only 77%, which was unexpected. However, in Belgium, the annual screening reports since 2017 showed that 15% of CC cases were HPV negative [60]. Additionally, using PCR with GP6/GP5 HPV universal primers in 113 cervical cancer biopsies from Iranian women, a similar HPV-positive percentage rate was reported (78%) [61]. We plan to evaluate the negative CC samples using next-generation sequencing to determine whether they are infected by other genotypes of HPV not detected by the LA.

It is important to mention that HPVs 16 and 18 are the most commonly detected high-risk genotypes worldwide, accounting for approximately 75% of all cervical cancer cases [12]. In our study, the prevalence of these genotypes in all CC samples analysed (*n* = 96) was only 53.1% (38.5% for HPV16 and 14.6% for HPV18). A study performed in central Mexico reported a prevalence in CC samples of 34% for HPV16 and 5% for HPV18 [62]. Therefore, the currently available vaccines may not be as effective in Mexico, and special attention should be paid to different genotypes that are not covered by the vaccine. Concerning HPV coinfections, as visualized in Fig. 1b, Figs. 2 and 3 b, almost all HPV genotypes preferentially occurred as multiple infections in the open-population and CIN 1 groups, while more than 50% occurred as single infections in the CC group, especially when HPVs 16, 18, 45, 59 and 58 were detected. Similar results have been reported previously in a Mexican population, where the most prevalent genotype detected in a single infection was HPV59, followed by HPV51 and HPV45 [45]. Worldwide, genotypes 16, 18 and 45 have been detected at a higher rate as single infections [63].

Considering these results, regional data on the prevalence and genotypic distribution of HPVs are essential for estimating the impact of vaccines on cervical cancer and screening programmes. Vaccination programmes with a quadrivalent vaccine (HPV6, 11, 16 and 18) and bivalent vaccine (HPV16 and 18), which have been approved by the FDA, have been implemented in over 40 countries [64, 65]. However, in 2012, Serrano et al. described the potential impact of a nine-valent (9-HPV) vaccine against HPVs 6, 11, 16, 18, 31, 33, 45, 52 and 58, reporting that this vaccine could prevent almost 90% of CC cases worldwide [66]. Nevertheless, this vaccine confers no protection against other HPV genotypes frequently found in women in the present study (HPV 59, 66, 51, 39 and 56). Therefore, it is necessary to continue analysing the geographical distribution of HPV genotypes in Mexico and worldwide to design effective HPV screening systems and develop new HPV vaccines. This is increasingly important as new technologies are rapidly detecting new HPV genotypes that cannot be detected by commercial detection tests [67–70].

It is important to mention that a limitation of this study was the lack of histological or cytological diagnosis of the samples from the women in the open-population group.

## Conclusion

Precancerous lesions caused by persistent HR-HPV infections are early indicators of cervical cancer. In this context, one of the most important prevention strategies is the implementation of HPV vaccination programmes. Current vaccines, although protecting against some of the most common HPV genotypes, do not confer protection against other prevalent HPVs detected in this study (59, 66, 51, 39 and 56). Therefore, the use of any of these vaccines should be based on local epidemiological data to cover the variations in the prevalence of HPV infections among regions. The differences in HPV prevalence and genotypic distribution described in this study could have a potential impact on the design of new vaccines and screening programmes to facilitate prevention of CC in Mexico. On the other hand, it is necessary to promote knowledge among the female population and raise awareness about the use of hormonal contraceptives and tobacco smoking as risk factors for CC development, in addition to HPV infection.

## Supplementary Information

**Additional file 1 Figure S1**. Frequency of HPV genotypes found in the open-population group in different states of Mexico. The graphs show the frequency of different HPV genotypes detected by the linear array genotyping test in the states of Aguascalientes, Colima, Nayarit, Jalisco, Michoacan and Guanajuato. The pink colour indicates single infections, and the blue colour indicates multiple infections.

## Data Availability

The datasets generated and/or analyzed during the current study are not publicly available but are available from the corresponding author on reasonable request.

## Abbreviations

HPV: Human Papilloma Virus
CC: Cervical Cancer
LA: Linear Array
CIN 1: Cervical Intraepithelial Neoplasia Grade 1
SI: Single infection
MI: Multiple Infection
HR-HPV: High Risk Human Papilloma Virus
