# Supplementary figures and images for: High frequency of HPV genotypes 59, 66, 52, 51, 39 and 56 in women from Western Mexico
Source: BMC Infect Dis. 2020 Nov 25;20:889. doi: 10.1186/s12879-020-05627-x (PMC7690193; doi:10.1186/s12879-020-05627-x)

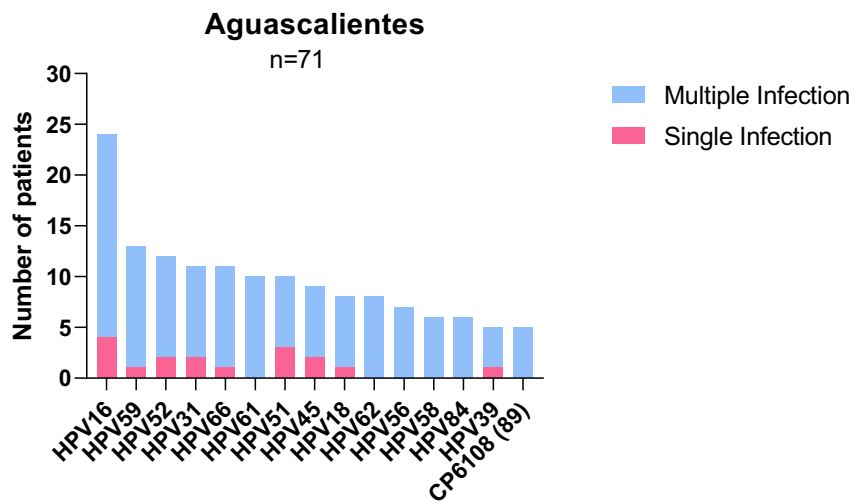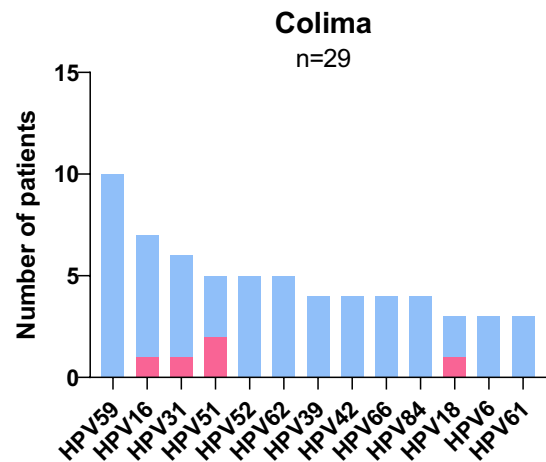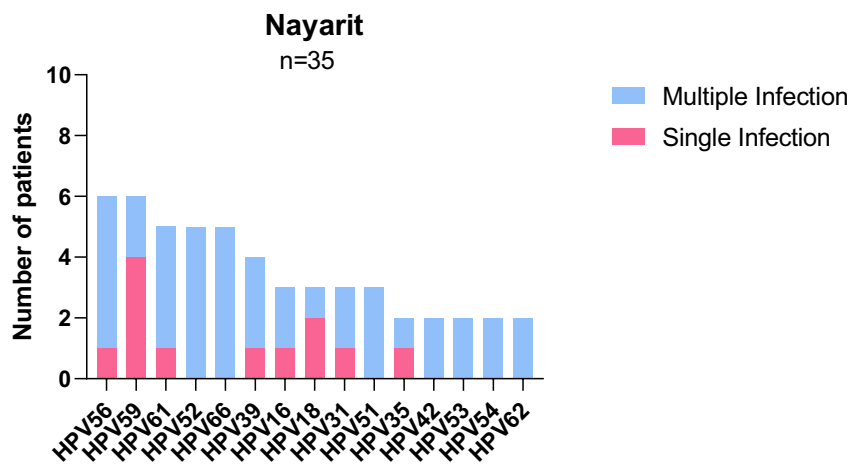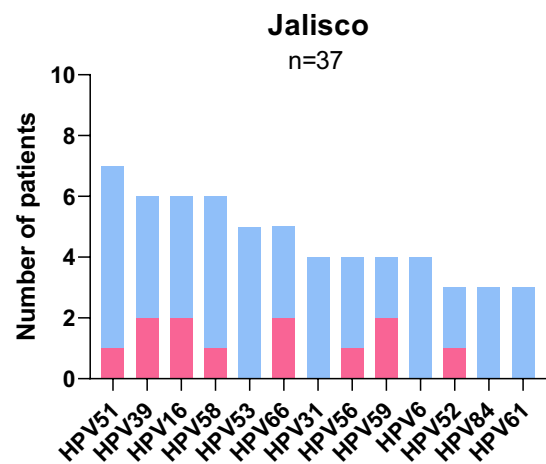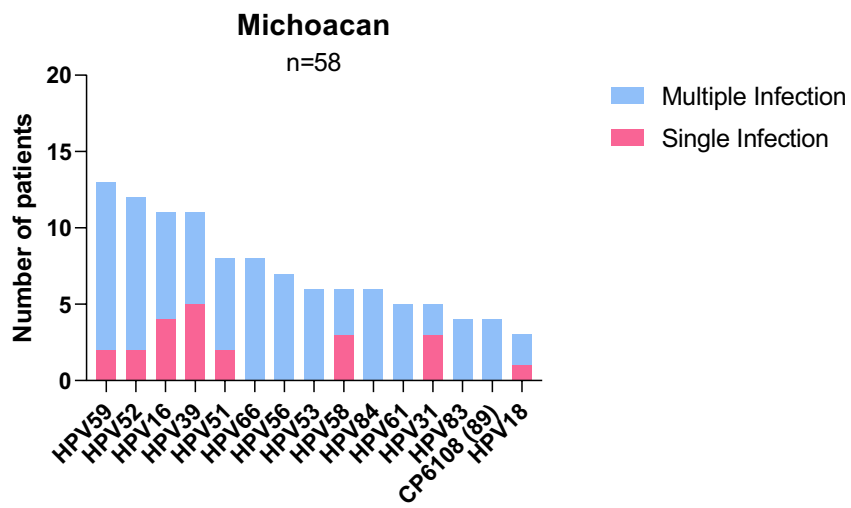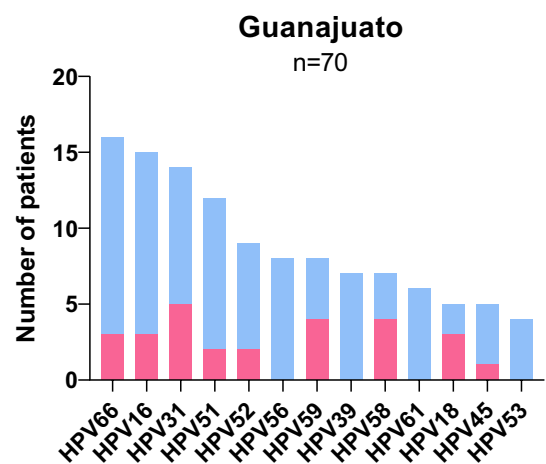

Supplement: Supplementary file 1 — Additional file 1 Figure S1. Frequency of HPV genotypes found in the open-population group in different states of Mexico. The graphs show the frequency of different HPV genotypes detected by the linear array genotyping test in the states of Aguascalientes, Colima, Nayarit, Jalisco, Michoacan and Guanajuato. The pink colour indicates single infections, and the blue colour indicates multiple infections. [file 12879_2020_5627_MOESM1_ESM.pdf]
